# Organic small-molecule-catalyzed carbonylation reactions

**DOI:** 10.1039/d6sc02732f

**Published:** 2026-04-28

**Authors:** Mao-Lin Yang, Le-Cheng Wang, Heifei Yang, Jiajun Zhang, Xiao-Feng Wu

**Affiliations:** a Leibniz-Institut für Katalyse e.V. Albert-Einstein-Str. 29a 18059 Rostock Germany Xiao-Feng.Wu@Catalysis.de xwu2020@dicp.ac.cn; b Dalian National Laboratory for Clean Energy, Dalian Institute of Chemical Physics, Chinese Academy of Sciences 116023 Dalian Liaoning China

## Abstract

C1 chemistry is important from both academic research and industrial application perspectives, and it is extensively studied with metal catalysts. Considering the advantages of organic small-molecule catalysts, it is highly attractive to explore their use as catalysts in carbonylation chemistry. This review examines organic small-molecule-catalyzed carbonylation reactions from an advanced catalysis perspective, with a particular emphasis on *N*-heterocyclic carbene (NHC) catalysis and oxygen, sulfur, selenium, nitrogen and phosphine main-group species-based catalysts to further expand the conceptual and synthetic landscape of sustainable carbonylation. These advances define carbonylation as a catalyst designed reaction and highlight opportunities for future organic small molecule driven development.

## Abstract 2

This review examines organic small-molecule-catalyzed carbonylation, with a particular emphasis on *N*-heterocyclic carbene (NHC), oxygen, sulfur, selenium, nitrogen, and phosphine main-group species-based catalysts.

## Graphical abstract

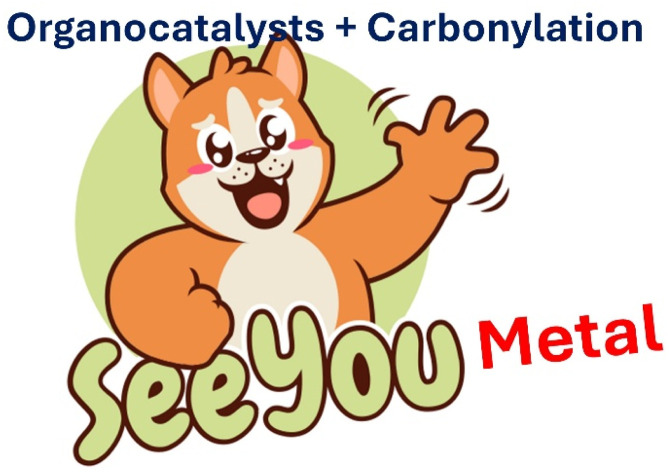

Carbonylation reactions constitute a powerful toolbox in modern synthetic chemistry because they provide a direct and atom-economic entry to carbonyl functionalities that are present in pharmaceuticals, natural products, agrochemicals and functional materials.^1^ According to the FDA statistics from 2015 to 2020, approximately 80% of approved drugs contain carbonyl functional groups (Fig. 1(1a)).^2^ For example, the widely prescribed anticoagulant Eliquis (ranked second in 2024 retail drug sales for thrombosis prevention) and the anti-myeloma agent Revlimid (ranked 28th) each contain three or more carbonyl groups and achieved market values of $20.699 billion and $5.809 billion, respectively.^3^ In synthetic chemistry, carbonylation technologies are among the most efficient and atom-economical methods for constructing carbonyl compounds;^4–8^ these processes convert carbon monoxide (CO)—derived from coal, natural gas or biomass—into value-added carbonylated products with essentially 100% atom utilization.^9^ CO is thus recognized as an abundant, cost-effective and versatile C1 feedstock in carbonylation chemistry. A canonical industrial example is methanol carbonylation to acetic acid, embodied by the Monsanto and Cativa processes, which together underpin the production of a substantial fraction of the world's acetic acid.^10,11^

**Fig. 1:**
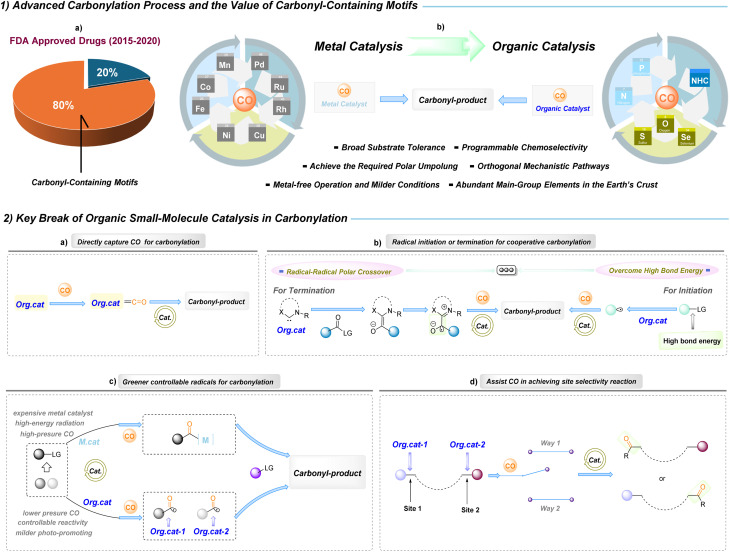
Background of organic small-molecule-catalyzed carbonylation reactions.

Since the early twentieth century, landmark metal-catalysed carbonylation processes, such as the Mond–Langer,^12^ Gattermann–Koch^13^ and Roelen oxo^14^ reactions, along with Heck's palladium-based methodologies^15^ and the multitude of transformations^4–8,16,17^ established a metal-centred paradigm in the field. These metal-based manifolds deliver exceptional efficiency and have industrial relevance, but they also impose intrinsic limitations: catalyst inhibition through strong metal–carbonyl binding, dependence on precious or toxic metals and specialised ligands, restricted substrate scope and diminished adaptability for late-stage functionalization of complex molecules. Moreover, metal-bound intermediates are prone to β-H elimination, isomerization and other deleterious pathways that minimize yield and selectivity. The importance of organic small-molecule catalysis was recognized by the Nobel Prize in Chemistry in 2021.^18^ Against this backdrop, organic small-molecule catalysis offers a complementary paradigm that directly addresses these bottlenecks while expanding the carbonylation design space. Rather than relying on metal–carbonyl coordination, organocatalytic strategies embed carbonylative reactivity within catalyst architecture and reaction design.^19–22^ Strongly coordinating organic motifs, sterically tuned molecular structures, and photo-activated organic mediators can each orchestrate the capture, transfer and transformation of carbonyl units. These approaches afford clear practical advantages—metal-free operation that eliminates residual contamination, cost-efficient and ligand-independent catalysts, milder and safer conditions, and broader compatibility with functionally complex substrates. Mechanistically, organocatalysis opens orthogonal polar and radical manifolds^19^ and enables controllable site-selectivity without recourse to special ligands; furthermore, reliance on abundant main-group elements enhances sustainability.^23^ Collectively, these features make organic small-molecule-catalyzed carbonylation particularly attractive for late-stage functionalization and for the environmentally responsible, sustainable development of CO-conversion processes (Fig. 1(1b)).

## Key breakthroughs in organic small-molecule-catalyzed carbonylation

Organic small-molecule-catalyzed carbonylation represents a mechanistically diverse platform. Current advances can be grouped into four key breakthroughs: controllable radical generation for carbonyl incorporation; organocatalytic-assisted CO activation to achieve site selectivity; direct capture of CO for carbonyl-bond construction; and cooperative manifolds that merge radical initiation or termination with organocatalytic carbonylation (Fig. 1(2a–d)).

### Direct capture and activation of CO

Nucleophilic or lone-pair-bearing organic catalysts (for example, carbenes or heteroatom donors) can engage CO by *σ*-donation and electronic polarization to produce acyl- or ketene-type adducts. Such capture transforms CO from an inert gas into a bound, activated intermediate that can be funnelled into downstream bond-forming steps without reliance on metal π-back bonding (Fig. 1(2a)).

### Radical initiation and termination in cooperative manifolds

Organocatalysts that enable polar Umpolung or generate persistent radical cations/anions can act as programmed initiators or terminators in radical carbonylation sequences. Soft, highly polarizable donors create directional charge transfer into σ*(C–X) orbitals, lowering the apparent bond dissociation energies and permitting selective cleavage to give otherwise inaccessible radicals; conversely, catalyst-promoted radical species can selectively trap carbonylated intermediates to close cooperative catalytic cycles (Fig. 1(2b)).

### Greener, controllable radical generation

Selective radical formation is typically accomplished through the formation of ground-state electron donor–acceptor (EDA) assemblies whose geometry, donor strength and electronic coupling determine which substrate undergoes charge transfer and subsequent homolysis. By tuning donor/acceptor properties and steric complementarity, organocatalysts can bias the EDA charge-transfer event toward the generation of specific, desired radical intermediates (Fig. 1(2c)).

### Organocatalyst-assisted site selectivity

Organocatalysts can pre-organize substrate-CO assemblies and modulate the redox landscape of the reaction environment, thereby biasing the position and timing of carbonyl incorporation. Differences in EDA geometry and the redox potential of the donor fragment change the kinetics and thermodynamics of key electron-transfer or polar steps, which, in turn, dictate reaction site-selectivity (Fig. 1(2d)).

These paradigms are not mutually exclusive: contemporary organocatalytic carbonylation often operates at the intersection of polar, radical and cooperative manifolds, reflecting increasingly sophisticated catalyst design. Collectively, they exemplify a central theme of modern carbonylation chemistry, that sustainable chemistry and catalyst-encoded control over electronic organisation and reaction topology can determine the identity, reactivity and fate of carbonylative intermediates, thereby expanding both the conceptual and practical boundaries of carbonylation.

## *N*-Heterocyclic carbene (NHC)-catalyzed carbonylation

The conceptual origins of Umpolung catalysis date back to the cyanide-catalysed benzoin condensation first reported in 1832,^24^ followed by Lapworth's formulation of the cyanide enolate mechanism in 1903.^25^ Early manifestations of polarity inversion in organocatalysis were closely associated with carbene chemistry, although imidazolylidene-type carbenes initially played only a minor role. A foundational contribution was made by Ukai and co-workers,^26^ who demonstrated that thiazolium salts can catalyse the benzoin reaction. This discovery was subsequently unified by Breslow's mechanistic insight,^27^ which identified a key nucleophilic intermediate formed between a carbene precursor and an aldehyde—now known as the Breslow intermediate—thereby establishing *N*-heterocyclic carbene (NHC) catalysis as a general strategy for polarity inversion. Efforts to introduce asymmetry into benzoin-type reactions were initiated by Sheehan and Hunneman in 1966 (ref. 28) with chiral thiazolium pre-catalysts. The field then underwent rapid expansion following Arduengo's isolation of stable carbenes,^29^ which enabled the development of structurally diverse NHC families, including triazolylidenes as well as annulated and mesoionic carbenes. These advances substantially broadened the steric and electronic landscape accessible to carbene catalysis. In parallel, mechanistic investigations revealed that Breslow intermediates are not confined to two-electron pathways but can also participate in single-electron-transfer processes. This reactivity mode was exemplified by Studer's biomimetic NHC-catalysed oxidation of aldehydes and further extended through radical and radical–polar crossover transformations reported by multiple research groups.^30^ In 2019, Nagao and Ohmiya introduced thiazolium-NHC-catalysed decarboxylative alkylation,^31^ a strategy that was soon adapted to multicomponent coupling reactions. Despite these substantial advances in both polar and radical NHC catalysis, the deliberate application of NHCs to carbonylation chemistry has remained relatively limited.

**Fig. 2:**
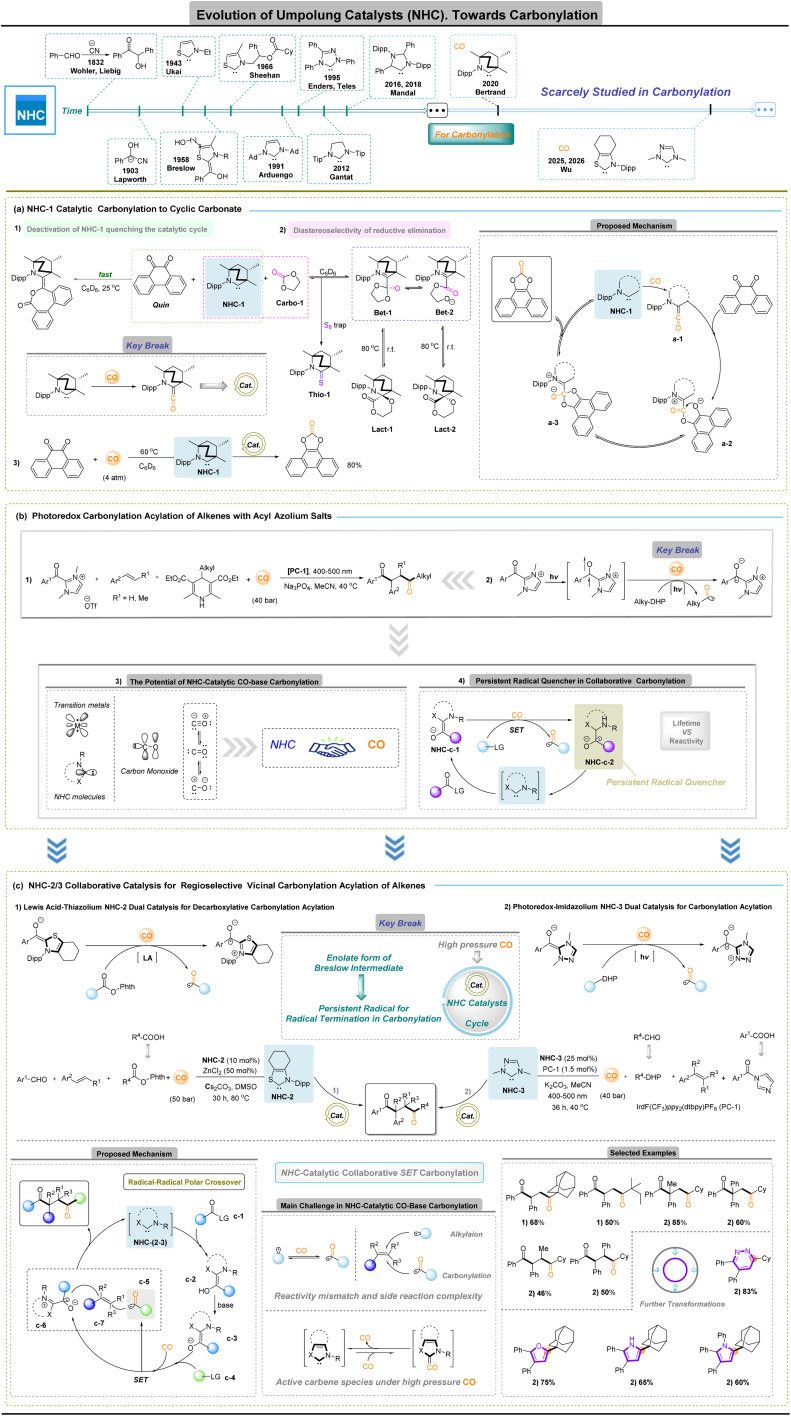
*N*-Heterocyclic carbene (NHC)-catalyzed carbonylation reactions.

A notable milestone was achieved in 2020, when Bertrand and co-workers reported the first example of CO-based NHC-catalytic carbonylation.^32^ In this study, a stable singlet ambiphilic carbene was shown to activate carbon monoxide and catalyse the carbonylation of *o*-quinone to afford cyclic carbonates, despite potential deactivation pathways such as *o*-quinone coupling in the reaction system (Fig. 2a-1). To gain insight into the diastereoselectivity of this process, NHC-1 was reacted with ethylene carbonate (Carbo-1) in C_6_D_6_ at room temperature. ^1^H NMR analysis revealed the formation of a 74 : 26 mixture of the diastereomers Lact-1 and Lact-2 (Fig. 2a-2). Upon heating the mixture to 80 °C, the diastereomeric ratio was reversed, favoring the thermodynamically more stable Lact-2. Furthermore, treatment of this mixture with elemental sulfur at 80 °C led to the formation of Thio-1 and regeneration of Carbo-1. Importantly, these observations strongly suggest that, unlike in organometallic catalysis, the reductive elimination step proceeds through a polar, stepwise mechanism involving open (Bet-1) and closed (Bet-2) betaine intermediates. The observed diastereoselectivity in reactions with *o*-quinone is therefore attributed to the superior leaving group ability of the phenoxy anion, which facilitates the formation of open betaine intermediates and consequently suppresses the accumulation of kinetic diastereomers. These findings also demonstrate the feasibility of closing the catalytic cycle using carbon monoxide. Overall, this work marks a key breakthrough, providing compelling evidence that the stable singlet ambiphilic NHCs are capable of controlled CO activation and utilization without compromising catalytic reactivity.

A growing and practically important paradigm in organocatalytic carbonylation is cooperative carbonylation, wherein NHCs need not act as the primary CO-activating species but instead regulate the reactivity, stabilization and selective termination of carbonylative intermediates generated elsewhere in the network. Within this framework, radical manifolds thus operate synergistically with classical Umpolung catalysis, expanding accessible reactivity while mitigating NHC deactivation.^32–34^ The approach is nevertheless constrained by the intrinsic affinity of NHCs for CO: strong *σ*-donation can sequester the carbene as ketene complexes, whereas productive turnover demands a long-lived acyl or persistent radical capable of intercepting carbonylated intermediates before carbene degradation. Balancing efficient CO engagement, radical lifetime and carbene stability therefore constitutes the central mechanistic challenge for NHC-based cooperative carbonylation.

**Fig. 3:**
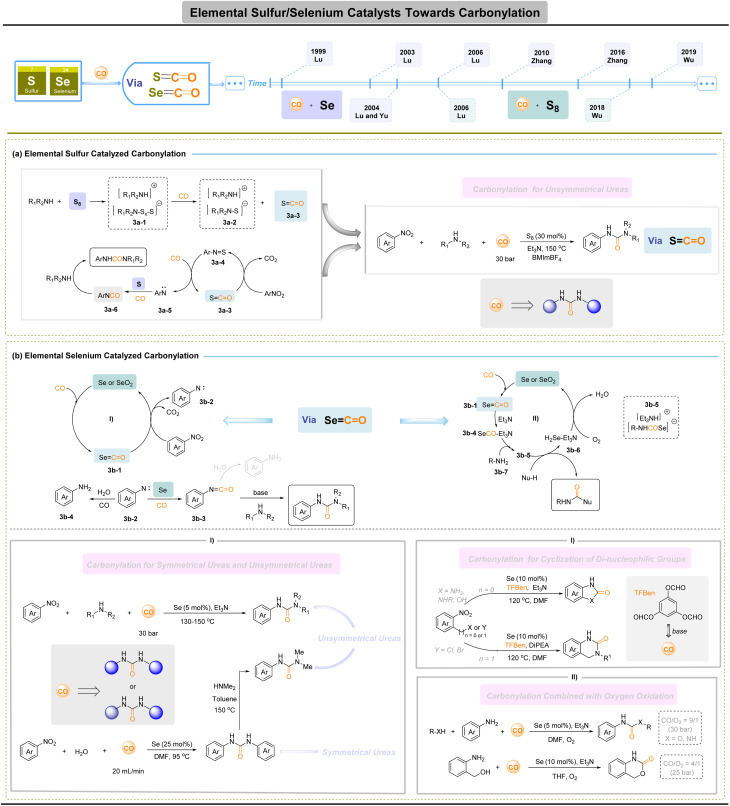
Elemental sulfur-/selenium-based catalysts for carbonylation.

**Fig. 4:**
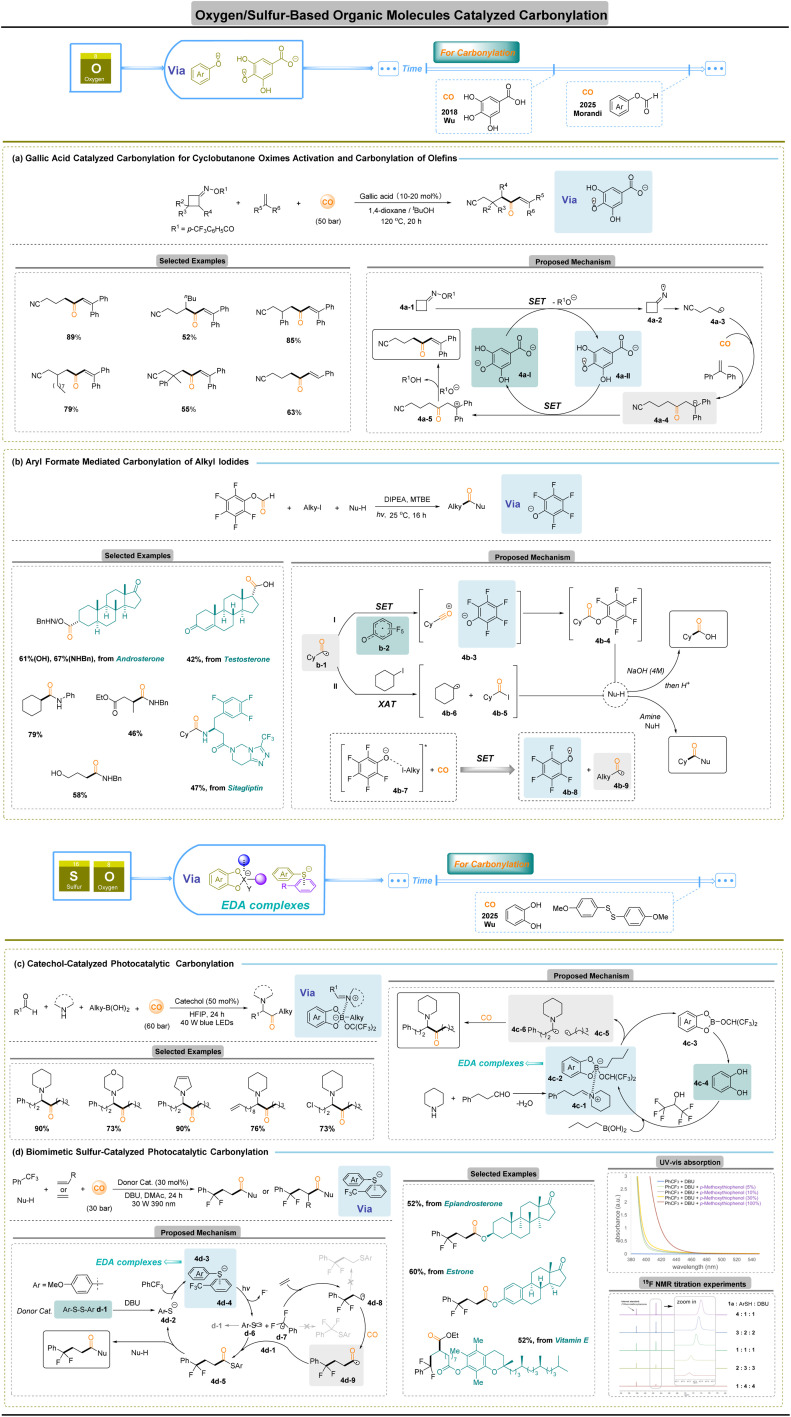
Oxygen-/sulfur-based organic small-molecule-catalyzed carbonylation.

In 2025, Wu and co-workers provided a persuasive demonstration of the cooperative NHC-radical carbonylation concept, delivering several advances that substantially expand the scope of NHC-based carbonylation chemistry. First, they established that persistent acyl radicals generated through CO-inclusive single-electron-transfer capture can be produced in a controlled manner and function as competent termination partners in carbonylative sequences; their diacylation of alkenes with acyl-azolium precursors to afford 1,4-diketones represents the first unambiguous example of persistent-radical-mediated carbonylative termination (Fig. 2b-1–4).^35^ Second, they realized NHC-catalysed carbonylation under high-pressure CO by integrating radical generation and efficient CO capture with classical acyl-azolium/Breslow reactivity, thereby demonstrating, for the first time, the applicability of thiazolium-^36^ and triazolium-^37^ based NHC catalysts in CO carbonylation (Fig. 2c-1 and 2). A mechanistically illuminating feature of the cooperative NHC-radical platform is the presence of a radical–radical polar-crossover manifold. Base activation of the carbene precursor generates the NHC-(2–3), which converts the acyl donor into a Breslow intermediate c-2 and its corresponding reducing enolate c-3. Single-electron transfer between this enolate and the radical precursor forms an oxidized Breslow species c-6 and an alkyl radical that traps CO to deliver an acyl radical c-5 (easy go decarbonylation). Regioselective coupling of the two radical partners (c-5 and c-6) with the alkene c-7 affords the 1,4-dicarbonyl product while regenerating the NHC catalyst, completing an NHC-catalysis, redox-neutral carbonylation manifold. This radical–radical polar-crossover logic reconciles the high reactivity of radical intermediates with the chemo- and regio-selectivity demands of catalytic carbonylation that couple controlled acyl radical formation with Breslow-type oxidation to deliver 1,4-diketones and related products. The resulting 1,4-diketones function as versatile C1 synthons, enabling C–C bond formation at sterically congested sites and direct access to diverse heterocycles beyond the reach of traditional C–H activation.

Collectively, these findings highlight two complementary roles for NHCs in carbonylation chemistry: (i) a direct, engineerable ability to engage CO and generate ketene-type intermediates, and (ii) a selective termination function that channels orthogonally formed acyl radicals into defined polar products. The present scarcity of NHC-catalysed CO-inclusive carbonylation thus marks not a limitation but an inflection point: with purpose-built carbene architectures and the deliberate integration of radical and relay concepts, the field is poised to evolve toward broadly applicable, genuinely sustainable carbonylation technologies capable of reshaping how carbonyl bonds are forged in complex molecule synthesis.

## Elemental sulfur-/selenium-based catalysts for carbonylation

Ureas are privileged motifs in agrochemicals and pharmaceuticals,^38–40^ yet their industrial synthesis typically relies on phosgene-derived routes, posing serious safety and environmental liabilities. Reductive carbonylation of nitro compounds has therefore emerged as an attractive non-phosgene alternative, largely enabled by noble-metal catalysts.^41–43^ The molecular electronic structure of CO, a polarized C≡O bond with accessible π* orbitals, makes it amenable to direct engagement by lone-pair donors. In this respect, main-group chalcogens, such as sulfur and selenium, offer a complementary, non-metal platform: their reversible redox manifolds (S: −2 to +6; Se: −2 to +4/+6) and accessible lone pairs enable diverse bond-activation modes and have long been exploited in organic synthesis.^44–46^ This valence flexibility allows S/Se species to capture, transfer, and release carbonyl fragments while using low-toxicity feedstocks. Expanding direct CO capture by these chalcogens to allow formation of ureas, through the controlled installation of two distinct amino groups in a metal-free carbonylative sequence, offers a practical and sustainable route to replace phosgene-based processes.

Early and foundational studies by Horwitz,^47^ Ohta,^48^ Bolze^49^ and Harper^50^ established the reactivity landscape of elemental sulfur with CO and related reagents, framing sulfur as a practical reagent and mediator for C–O and C–N bond construction under relatively simple conditions (Fig. 3a). Representative catalytic implementations illustrate both the promise and the distinct mechanistic logic of S-catalytic carbonylation. In 1996, Macho and co-workers reported that elemental sulfur,^51^ in the presence of NaVO_3_ and methanol under high CO pressure (≈13 MPa), converted nitrobenzene predominantly to methyl *N*-phenylcarbamate (MPC), implicating sulfur-promoted formation of activated carbonyl intermediates. Subsequent work by Lu and colleagues (2006) extended this concept to the oxidative–reductive carbonylation of nitroaromatics and anilines in ionic liquids, delivering unsymmetrical ureas.^52^ Mechanistic studies converge on a common motif: elemental sulfur reacts with amines and CO for the *in situ* formation of thiolate-type salts 3a-1, which then generate COS (S=C=O) 3a-3; COS then facilitates the deoxygenation of nitroarenes to nitrene-type intermediates 3a-5, which are intercepted by S and CO to furnish aroyl isocyanates (ArNCO) 3a-6 that are ultimately trapped by amines to give unsymmetrical ureas. Sulfur's intrinsic advantages, including facile redox interconversion, ready availability (including industrial by-products), low toxicity and alignment with resource-circularity principles, make it a compelling platform for sustainable carbonylation.

Elemental selenium constitutes a main-group platform for metal-free carbonylation that is closely related to sulfur chemistries. Owing to its accessible lone pairs and flexible redox manifold, Se can form reactive SeCO species *in situ* and thereby mediate carbonyl transfer under phosgene-free conditions. Early and sustained efforts, from Franz's^53^ and Sonoda's^54^ seminal studies through more recent methodological developments, have translated these fundamental properties into practical protocols for preparing carbamates, thiocarbamates, carbonates, and both symmetric and asymmetric ureas, demonstrating that Se/CO systems provide operationally simple alternatives to phosgene-based chemistry.

Two mechanistic manifolds dominate Se-catalysed carbonylation. In the reductive carbonylation route (Fig. 3b-I), elemental Se reacts with CO (and base) to generate SeCO intermediate 3b-1 that effect deoxygenation of nitroarenes to nitrene-like species 3b-2. These nitrenes are then converted, *via* Se/CO chemistry, into aroyl isocyanates b-3 or related acyl transfer agents that are intercepted by amines 3b-4 to give ureas. A series of reports (Lu *et al.*, 1999;^55^ 2003;^56^ 2004;^57^ 2006 (ref. 58) and Zhang *et al.*, 2016 (ref. 59)) exemplify this logic, delivering both unsymmetrical and symmetrical ureas in one-pot, phosgene-free sequences. These reductive Se cycles have also been adapted for intramolecular cyclizations of di-nucleophilic substrates (*e.g.* Se-promoted syntheses of dihydroquinazolinones^60^ and benzimidazolones^61^), illustrating the manifold synthetic utility of Se-catalytic CO transfer. The second principal pathway (Fig. 3b-II) is oxidative (O_2_-mediated) Se catalysis, developed notably by Zhang and co-workers (2010–2018). Here, Se/CO chemistry is merged with an external oxidant to effect oxidative carbonylations of amines and alcohols, enabling one-pot access to *N*-phenylcarbamates,^62^ benzoxazinones^63^ and heterocyclic urea derivatives^64^ under mild conditions. Typical cycles invoke *in situ* generation of SeCO 3b-1, nucleophilic addition to form phenylcarbamoselenoic Se-acid salt 3b-5 from carbonyl selenide 3b-4, and nucleophilic displacement to yield the carbonyl product, accompanied by the formation of hydrogen selenide 3b-6. Hydrogen selenide was then oxidized to selenium by oxygen for the upcoming catalytic cycle. Selenium offers parallel practical benefits as a phosgene-free route to ureas, carbamates and related motifs.

## Oxygen-/sulfur-based organic small molecules for carbonylation

Organic molecule radicals are indispensable intermediates in synthesis, yet their catalytic exploitation remains limited, and their generation has traditionally relied on specialized initiation strategies. Although photo/electrochemistry catalysis has transformed radical chemistry over the past decade,^65–67^ the prevailing reliance on metal complexes raises concerns over cost.^63–67^ These limitations have stimulated intense interest in metal-free radical carbonylation, which offers a sustainable alternative by avoiding precious metals.

One early example is the 2018 report by Wu and co-workers (Fig. 4a),^68^ in which they used a gallic acid-derived catalyst to trigger a ring-opening carbonylation of cyclobutanone oxime esters. In this system, the deprotonated gallic acid 4a-I single-electron transfers to the oxime 4a-1, producing an iminyl radical 4a-2 and a gallic ester radical a-II. The iminyl radical 4a-2 undergoes rapid β-scission to give a distal cyanoalkyl radical a-3, which promptly captures CO to form the acyl radical intermediate 4a-4, producing the ketone product. This neat mechanism uses a biomimetic oxygen donor (gallic acid) to catalyse the SET and ring opening, illustrating how an organic acid can replace a metal catalyst. More recently, Morandi and co-workers reported a metal- and CO-free carbonylation of alkyl iodides under photochemical conditions (Fig. 4b).^69^ Their strategy employs a simple aryl formate as a green bifunctional reagent that serves simultaneously as a CO source and a mediator for the carbonylation process. Upon visible-light irradiation, the phenolate acts as a photoredox donor, reducing the alkyl iodide to an alkyl radical while generating a phenoxy radical 4b-2. The alkyl radical then adds to the *in situ* CO to give an acyl radical 4b-1, which is oxidized by the phenoxy radical 4b-2 to an acylium–phenolate ion pair 4b-3; recombination of this ion pair furnishes the carboxylic acid or amide product (pathway I). Alternatively, an acyl iodide 4b-5 arising from a halogen-atom transfer (XAT) between acyl radical 4b-1 and alkyl iodide would also lead to target products upon nucleophilic substitution (pathway II). Importantly, this process “overcomes the typical need for CO gas and/or metal catalysts”. Mechanistic studies (including preparation of putative intermediates and measurement of a very low quantum yield, *Φ* ≈ 0.001) indicate a closed SET pathway rather than a radical chain mechanism. Overall, the Morandi method is highly efficient and versatile, tolerating a wide variety of functional groups, despite relying solely on bench-stable organic reagents (aryl formate and base). Altogether, these results support the conclusion that pathway I is more likely under their reaction conditions.

Electron donor–acceptor (EDA) complexes activate otherwise inert bonds through ground-state charge-transfer assemblies whose visible-light excitation induces single-electron transfer to generate radical-ion pairs.^70–72^ This pre-polarization lowers the homolysis barrier, enabling carbon-centred radicals to capture CO rapidly and form acyl radicals for acylation and carbonylative difunctionalization under mild, metal-free conditions. Key challenges remain, including back-electron transfer that limits radical lifetimes, reversible CO capture, and the need for precise control of donor–acceptor geometry and redox matching to ensure selectivity. Modular organic small molecules offer a solution: their tunable electronic and steric features allow programming of charge-transfer strength and radical reactivity, transforming EDA chemistry into a general, designable platform for metal-free carbonylation across diverse bond classes.

C–B bonds exhibit a moderate bond strength (76–79 kcal mol^−1^),^73^ and organoboron species are readily converted into alkyl radicals through photo-induced SET within EDA complexes. This feature renders alkylboronic acids exceptionally versatile partners for radical carbonylation. In 2025, Wu and co-workers disclosed a catechol-catalyzed four-component reaction of alkylboronic acids, aldehydes, amines, and CO to access α-amino ketones (Fig. 4c).^74^ In this process, ^*n*^butylboronic acid forms boronate 4c-2 with catechol, while condensation of aldehyde and amine affords iminium 4c-1; these assemble into an EDA complex in HFIP. Photoexcitation generates radicals 4c-5 and 4c-6, and hydrolysis of intermediate 4c-3 regenerates catechol 4c-4. Radical 4c-5 undergoes CO insertion to an acyl radical that couples with 4c-6 to yield the product. The transformation highlights how tailored oxygen donors enable controlled C–B activation and rapid CO capture in a catalytic cycle.

Activation of Ar-CF_3_ units represents one of the most demanding challenges in radical chemistry owing to the exceptional strength of C–F bonds (109 kcal mol^−1^)^75^ and the propensity of partially defluorinated intermediates toward over-reaction. EDA complexes offer a means to polarize these inert bonds *via* charge-transfer excitation. A recent biomimetic sulfur-catalyzed strategy demonstrated by Wu and co-workers enables carbonylative difunctionalization of unactivated trifluoromethylarenes (Fig. 4d).^76^ The arylthio anion and trifluorotoluene form an EDA complex; photo-SET produces the sulfur radical 4d-6 and a trifluorotoluene radical anion, which expels fluoride to afford difluorobenzylic radical 4d-7. Addition of 4d-6 to alkenes gives the alkyl radical 4d-8, which captures CO to give the acyl radical 4d-9. Intermediate 4d-5 arises either through cross-coupling of 4d-9 with sulfur radical d-6 or radical substitution on disulfide. Subsequent nucleophilic acyl transfer releases the product and regenerates the thioanion catalyst. This work establishes EDA-mediated C–F cleavage as a viable platform for carbonylation of previously inaccessible fluoroalkyl feedstocks.

Together, these platforms combine sustainable operation, bio-derived catalysts and modular donor design, yet remain constrained by back-electron transfer, reversible decarbonylation and limited catalytic turnover. Moving forward, mechanism-guided tuning of organic electronic donor catalysts and integration with photochemical or XAT relays will be pivotal for transforming these concepts into broadly applicable carbonylation technologies.

## Phosphorus-based organic small-molecule-catalyzed carbonylation

Significant progress has been made in the field of C–X carbonylation in recent decades, with most advances involving transition-metal-catalyzed processes.^77–83^ Nevertheless, a general protocol for the carbonylation of organohalides with CO under mild conditions is still lacking. Major obstacles include limited functional-group tolerance and competitive side reactions, such as over-reduction and hydrodehalogenation, arising primarily from the highly negative reduction potentials of halides. Alkyl radicals readily add to CO to form acyl radicals, but this step is reversible and decarbonylation is thermodynamically favoured, such that conventional protocols typically demand high CO pressures (>40 bar) to sustain productive turnover.

**Fig. 5:**
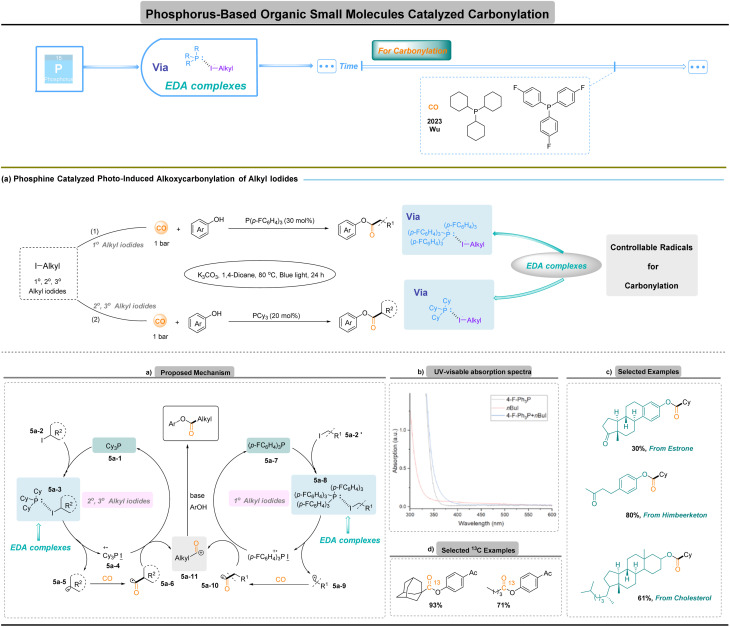
Phosphorus-based organic small-molecule-catalyzed carbonylation.

In 2023, Wu and co-workers revealed that organophosphines can function as catalytic activation platforms rather than simple nucleophiles (Fig. 5):^84^ under visible light, electron donor–acceptor (EDA) complexation between a phosphine and an alkyl iodide triggers single-electron transfer to generate alkyl radicals that capture CO efficiently at 1 bar. This metal-free manifold circumvents the need for high CO pressure and avoids the poisoning, ligand dependence and functional-group restrictions associated with transition metals, while enabling reagent-controlled selectivity through the choice of phosphine donor. A notable and mechanistically informative feature of the system is that the choice of phosphine determines which class of alkyl radical is generated and therefore which substrates are efficiently carbonylated (Fig. 5a). Tris(4-fluorophenyl)phosphine 5a-7 selectively forms an EDA complex 5a-8 with primary alkyl iodides 5a-2′ and promotes cleavage to primary alkyl radicals 5a-9, while tricyclohexylphosphine 5a-1 preferentially activates secondary and tertiary alkyl iodides 5a-2 to give corresponding radicals 5a-5*via* EDA complex 5a-3. UV-visible spectroscopy confirms EDA complex formation through a characteristic red shift upon mixing phosphine and alkyl iodide, and mechanistic studies support a pathway in which blue-light excitation of the EDA complex effects charge transfer to generate a phosphine radical cation and an alkyl radical that engages CO (Fig. 5a and b). This ligand-controlled radical selectivity translates directly into substrate scope control: primary, secondary and even sterically hindered tertiary radical precursors can be engaged simply by changing the phosphine, an operationally simple lever that is not readily available in conventional metal-catalysed carbonylation.^85^

In contrast to earlier protocols that required high-energy Xe lamps or metal catalysis, current protocols proceed efficiently under low-energy irradiation. The resulting methodology offers practical advantages: reactions operate at atmospheric CO pressure, minimizing engineering demands and safety risks associated with high-pressure equipment, and the mild conditions translate into broad functional-group tolerance. The platform is also well-suited to isotopic labelling, enabling synthesis of ^13^C-labelled phenyl esters under 1 bar CO with minimal isotope waste, which is especially valuable for radiochemistry and drug-metabolism studies (Fig. 5c and d). Overall, phosphine-mediated photoalkoxycarbonylation merges radical CO capture with the tunability of organocatalysts, providing a versatile complement to classical metal-mediated carbonylation for the selective incorporation of CO into complex molecules.

## Nitrogen-based organic small-molecule-catalyzed carbonylation

Carbonylation of site-selective aryl C–H^86–89^ with alkyl halides^81–83^ or some nucleophilic reagents *via* radical manifolds has historically been constrained by three interrelated problems: high bond-dissociation energies of the parent substrates, the intrinsic instability of acyl radicals (which favor decarbonylation) and the highly negative reduction potentials of C(sp^3^)–halogen bonds (*E*_red_ ≪ −2.0 V *vs.* SCE), which hinder mild, metal-free activation. Recently, Wu and co-workers addressed both challenges by exploiting multifunctional electron-donor–acceptor (EDA) assemblies in which a single organic molecular mediated manifold is programmably repurposed to generate distinct reactive intermediates and termination modes.^90,91^

**Fig. 6:**
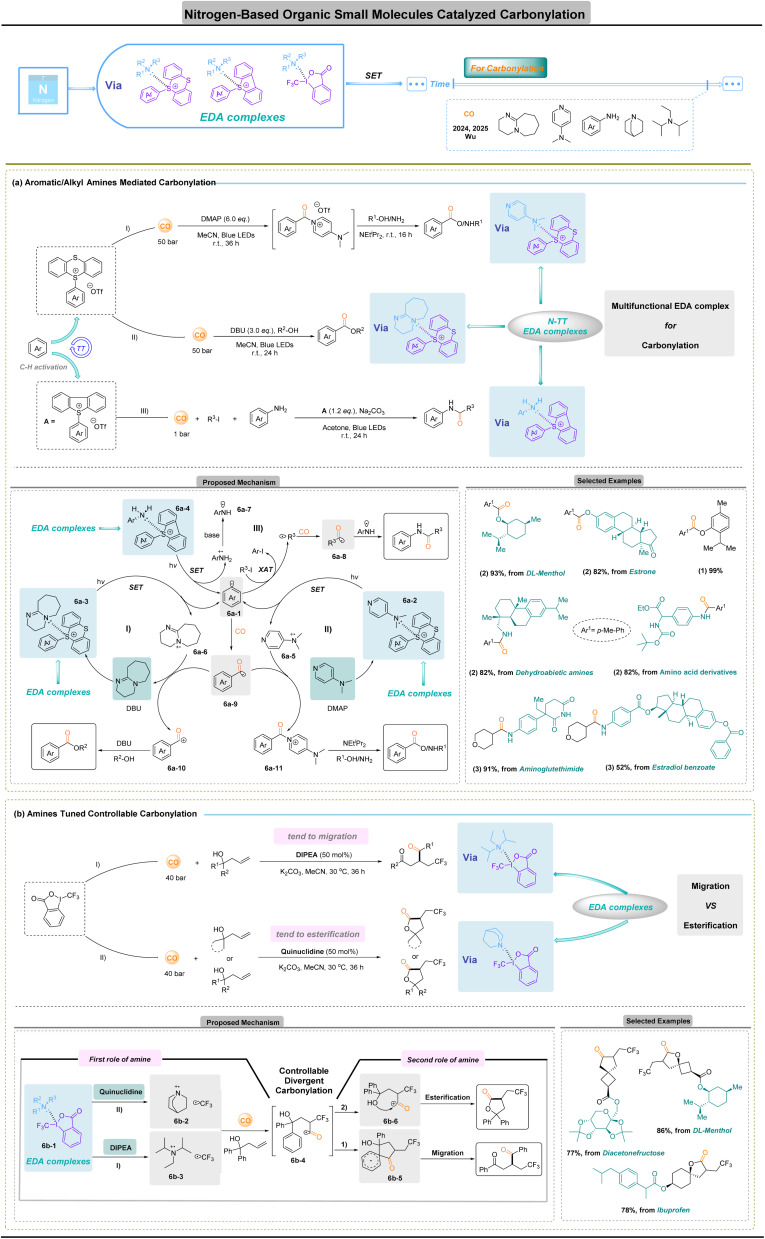
Nitrogen-based organic small-molecule-catalyzed carbonylation.

In one implementation (Fig. 6a-I and II),^90^ aryl thianthrenium (TT) salts form EDA complexes 6a-2 and 6a-3 with tertiary amines; visible-light excitation produces aryl radicals 6a-1 that capture CO to give acyl radicals 6a-9, and the downstream fate of these intermediates is dictated by the amine partner. Strong, non-nucleophilic donors (*e.g.* DBU, Fig. 6a-II) bias the sequence toward single-electron oxidation of the acyl radical and formation of acylium-type species 6a-10, whereas nucleophilic donors (*e.g.* DMAP, Fig. 6a-I) deliver stabilized aroyl–amine adducts 6a-11 that engage a wide range of O- and N-nucleophiles. This conditional reactivity permits selective, metal-free C–H conversions to esters and amides under mild, visible-light conditions and with broad functional-group compatibility. Complementary advances extend the concept to alkyl substrates by merging EDA photochemistry with halogen-atom-transfer (XAT) logic (Fig. 6a-III).^91^ Aryl sulfonium/phenyl-radical generation *via* aniline–sulfonium EDA 6a-4 excitation produces an aryl radical 6a-1 that effects XAT on otherwise intransigent alkyl iodides to give alkyl radicals; these species trap CO to form acyl radicals 6a-8 that couple with nitrogen-centred fragments to furnish amides under metal-free conditions. Critically, the XAT relay circumvents the need for direct, highly reducing single-electron transfers to C(sp^3^)–X bonds, thereby obviating transition-metal mediators and enabling efficient carbonylation despite unfavorable reduction potentials.

In these works, multifunctional EDA assemblies, programmed by simple donor (amine) choice or by incorporation of XAT relays, convert a common carbonyl precursor into distinct reactive manifolds, acylium ions, aroyl–donor adducts or acyl radicals, thereby enabling diverse, mild and metal-free carbonylative outcomes. By encoding selectivity in readily tunable organic donors rather than bespoke metal–ligand motifs, this approach improves functional-group tolerance and late-stage applicability and offers an operationally simple, scalable and sustainable complement to classical metal-based carbonylation.

Extending beyond simple product diversification, amine-driven EDA platforms enable site-selective carbonylation by encoding the fate of CO-derived acyl intermediates through the steric and redox properties of the donor.^92–94^ Nevertheless, achieving reliable regio- and chemo-selectivity remains a central challenge when a single substrate presents multiple competing reaction sites.^95–101^ Recent EDA photochemical strategies^70–72^ address this problem by exploiting tertiary amines such as dual-function components, electron donors that generate carbon radicals, and redox partners whose radical-cation forms steer subsequent reactivity.

A striking demonstration of this concept was reported in 2025 (Fig. 6b),^102^ where one substrate was steered selectively to either 1,4-diketones (Fig. 6b-I) or γ-lactones (Fig. 6b-II) solely by switching different tertiary amines. After rapid CO capture forms an acyl radical, a polarity bifurcation emerges: outer-sphere electron transfer (OSET) converts the acyl radical into an electrophilic acylium ion, whereas suppression of outer-sphere single-electron transfer (OSET) preserves the nucleophilic acyl radical. The donor's steric and electronic profile determines this divergence, dictating whether the intermediate undergoes intramolecular lactonization or remote aryl migration. Photoexcitation of an EDA complex 6b-1 between Togni reagent and the amine generates a CF_3_ radical and an amine radical cation; addition to an alkene followed by CO capture affords the key acyl radical 6b-4. Computations showed that, with quinuclidine, the OSET barrier to form the acylium species 6b-6 (≈5.1 kcal mol^−1^) is lower than the competing radical aryl-attack esterification pathway (≈7.3 kcal mol^−1^), leading to lactonization (Fig. 6b-II-2), whereas DIPEA raises the OSET barrier (≈8.3 kcal mol^−1^) and favours aryl migration *via*6b-5 to deliver 1,4-dicarbonyl products (Fig. 6b-I-1).

Conceptually, this manifold, which includes an amine catalytic donor-switchable EDA complex with CO, decouples activation from termination, encoding reaction-site selectivity in simple organic molecules rather than in bespoke metal–ligand architectures. In contrast to classical metal-catalyzed carbonylation, where selectivity is fixed at the metal activation stage and often compromised by β-H elimination, catalyst poisoning, and residual-metal concerns, the present strategy operates under mild conditions without transition metals, avoids ligand design constraints, and tolerates densely functionalized substrates. Notably, CO insertion extends the carbon chain and unlocks migration pathways that are otherwise inaccessible to short homoallylic frameworks.

Moreover, a base-promoted radical carbonylation of activated alkylamines with phenols and alcohols was developed by Wu and co-workers in 2021.^103^ The alkyl amines were activated by Katritzky salts, and the reaction proceeded well under low CO pressure (1–6 bar). Various desired esters were obtained in moderate to excellent yields.

## Conclusions and perspectives

In this review, we have expounded on the remarkable potential inherent in organic small-molecule catalysts to unlock mechanistic alternatives to classical metal-mediated carbonylation that carry distinct advantages for high-precision and sustainable chemistry. Whereas metal routes have been defined by metal–CO coordination, ligand-directed migratory insertion and the limitations those features impose (precious-metal cost, metal residues, ligand screening, β-H elimination, catalyst poisoning), organic donors, such as *N*-heterocyclic carbenes, chalcogenides (S/Se), and nitrogen-, phosphine-, and oxygen-based scaffolds, access alternative entry points: direct CO capture to give ketene- or acyl-type adducts, programmed single-electron manifolds (EDA/XAT) that generate radicals under visible light, and cooperative relay/termination cycles that convert radical events into defined polar products. These non-metal manifolds deliver immediate practical advantages (low-toxicity, low-cost catalysts, operation at ambient or low CO pressures and with bench-stable CO surrogates, visible-light compatibility, and enhanced functional-group tolerance) while opening genuinely new reaction spaces, for example, donor-encoded site-selectivity, late-stage acylation of densely functionalized molecules, and carbonylation strategies that are difficult or impossible under conventional metal catalysis.

Broader adoption of organic small-molecule carbonylation will depend on coordinated, question-driven research that tightly couples mechanistic insight with catalyst design and process development. Key research priorities fall into three complementary strands. Mechanistic imperatives include rigorous quantification and control of SET-manifolds (suppression of back-electron transfer, control of radical lifetimes and avoidance of reversible decarbonylation), and the physical-organic mapping of how donor–acceptor geometry, redox matching and medium effects determine productive CO capture. Platform maturation requires methodological consolidation: development of organic molecular-based catalytic redox cycles for main-group donors; systematic integration of EDA/XAT/photochemical and electrochemical activation modes; and optimization of organic donor scaffolds to maximize turnover, selectivity and reagent economy. Translational application calls for process-oriented designs, including scalable, flow-compatible protocols, safe low-pressure or surrogate CO development, isotope-label workflows, and curated research of donor–acceptor assemblies and redox parameters to enable predictive catalyst design.

Framing carbonylation as a catalyst-designed reaction class places organic small-molecule strategies at the intersection of mechanistic innovation and sustainable synthesis. By tailoring catalyst structure and electronics, organic donors can dictate when, where and how CO is engaged, thereby directing the formation, polarity and lifetime of acyl intermediates (*e.g.* acyl radicals *versus* acylium ions) and converting transient species into predictable, selectable outcomes. Coupled with the operational advantages of metal-free protocols (benign nature, low-cost catalysts, visible-light activation, ambient-pressure CO or benign surrogates and enhanced functional-group tolerance), this mechanistic control opens programmable routes to late-stage acylation and other carbonylative transformations that are difficult or impractical under classical metal catalysis. Given the clear convergence of conceptual novelty, practical utility, and sustainability, targeted research into catalyst design, catalytic redox cycling, integration of EDA/XAT/photochemical modalities, and process translation (flow, CO-surrogates, isotope workflows) is both timely and warranted.

## Author contributions

Mao-Lin. Yang: writing – original draft. Le-Cheng Wang: literature search. He-Fei Yang: literature search. Jia-Jun Zhang: literature search. Xiao-Feng Wu: writing – review & editing, supervision, funding acquisition, conceptualization.

## Conflicts of interest

The authors declare no competing interests.

## Data Availability

No primary research results, software or code have been included and no new data were generated or analysed as part of this review.
